# Diagnostic accuracy of the Dutch version of the 4AT for delirium detection in a mixed patient population and setting

**DOI:** 10.1007/s40520-023-02447-2

**Published:** 2023-06-07

**Authors:** Maaike A. Pouw, Agneta H. Calf, Rita R. Georg, Sophia E. de Rooij, Jan C. ter Maaten, Barbara C. van Munster

**Affiliations:** 1 Department of Geriatric Medicine, University of Groningen, University Medical Center Groningen, Hanzeplein 1, 9700 RB Groningen, The Netherlands; 2 Amstelland Hospital, Laan van de Helende Meesters 8, 1186 AM Amstelveen, The Netherlands; 3 Department of Internal Medicine, University of Groningen, University Medical Center Groningen, Hanzeplein 1, 9700 RB Groningen, The Netherlands

**Keywords:** Diagnostic test accuracy, Delirium, 4AT, Emergency department, Hospital

## Abstract

**Background:**

Delirium is an acute disturbance in attention, awareness and cognition. Immediate detection in older adults is recommended because delirium is associated with adverse outcomes. The 4 ‘A’s Test (4AT) is a short screening instrument for delirium. The aim of this study is to evaluate diagnostic accuracy of the Dutch version of the screening tool 4AT for delirium detection in different settings.

**Methods:**

Prospective observational study conducted in two hospitals in patients aged ≥ 65 years in geriatric wards and the Emergency Department (ED). Each participant underwent two assessments; the index test 4AT, followed by the reference standard for delirium performed by a geriatric care specialist. The reference standard delirium is according to the Diagnostic and Statistical Manual of Mental Disorders (DSM-V) criteria.

**Results:**

A total of 71 geriatric inpatients and 49 older ED patients were included. The prevalence of delirium was 11.6% in the acute geriatric ward and 6.1% in the ED. The sensitivity and specificity of the 4AT in the acute geriatric ward were 0.88 and 0.69, respectively. In the ED, the sensitivity and specificity were 0.67 and 0.83, respectively. The area under the receiver operating characteristic curve was 0.80 for the acute geriatric ward setting and 0.74 for the ED setting.

**Conclusion:**

The Dutch version of the 4AT is a reliable screening tool for delirium detection in both acute geriatric wards and ED. Due to its brevity and practicality (i.e., no special training is required to administer the tool), it is useful in clinical practice.

## Introduction

Delirium is an acute disturbance in attention, awareness and cognition, as a direct consequence of a physiological event such as an acute disease [1]. The prevalence and incidence of delirium in acute medical adult inpatients is about one in four older patients, and these numbers have remained broadly stable in the past decades [2]. The presence of delirium should prompt health care professionals to search for the underlying illness or cause and treat accordingly. Delirium is often a sign of vulnerability and is associated with adverse outcomes of hospitalization, including increased mortality, longer length of hospital stay, poorer functional status and institutionalization [3, 4]. Although the awareness of delirium is arising, the diagnosis of delirium is often overlooked, especially in settings such as an Emergency Department (ED) [5]. An active approach pays off as higher screen rates are associated with fivefold higher recognition rates, demonstrating the need for screening in clinical practice [4].

Various screening tools have been developed to identify patients with delirium in different settings. The 4A’s Test (4AT) was most promising for ruling out delirium in the ED in a systematic review and meta-analysis on screening instruments in the emergency department [6]. The 4AT is a brief screening tool developed and designed for the detection of delirium and comprises four items: level of alertness, the Abbreviated Mental Test-4 (AMT4), attention testing with the months backward and acute change or fluctuation in mental status [7]. It has a score range of 0–12, with scores of 4 or more (> 3) suggesting possible delirium [8]. It showed to be an applicable screening tool for use in clinical practice by different professionals and levels of seniority, is well tolerated by patients and only takes a few minutes to conduct [9].

A recent systematic review and meta-analysis on the performance of the 4AT in various settings; ea. ED, acute medical ward and care facility, showed good performance of the 4AT with pooled sensitivity and specificity of 0.88 and 0.88, respectively [10]. The aim of the present study is an evaluation of the diagnostic test accuracy of the Dutch version of the 4AT as a screening tool for delirium in older persons in two settings; ED and acute medical and geriatric ward. The Dutch version of the 4AT is evaluated against a reference standard based on DSM-V criteria for delirium.

## Methods

### Study design

This study complies to the Standards for Reporting of Diagnostic Accuracy Studies guidelines, the STARD, 2015 [11]. It is a prospective observational study conducted in two Dutch hospitals: (1) the University Medical Center Groningen (UMCG), a tertiary teaching hospital which serves both as an academic and a general hospital in the area, and (2) Gelre Hospitals, a general teaching hospital. The local Medical Research Ethics Committee of the UMCG waived the study, since it comprises an evaluation of routine clinical practice. Written informed consent was not required and verbal information and consent were sufficient.

### Study setting and sample

The study was conducted in two geriatric wards and the Emergency Department. Between February 13 and April 17 2018, in an acute medical geriatric ward of the UMCG and between January 9 and February 2, 2018 in the geriatric ward of the Gelre Hospital. Both these wards are staffed by experts in geriatric medicine (geriatricians and internist-geriatricians, i.e., internists with expertise in geriatrics) and have weekly grand rounds in which a team of residents and experienced geriatricians/internist-geriatricians systematically assesses the four geriatric domains (i.e., somatic, psychiatric, functional, and social domain), which include evaluation of the presence of delirium according to the American Psychiatric Association’s fifth edition of the *Diagnostic and Statistical Manual of Mental Disorders*(DSM-V) criteria [1].

Between April 4 and April 23, 2018, and between September 21, 2021 and March 30, 2022, a prospective, non-consecutive sample of patients presenting to the ED of the UMCG was recruited. In the latter period, data collection was included via Acutelines, a biobank that started in 2020 which collects data from patients presenting with acute conditions to explore the association between pre-existing health, acute illness and (long-term) outcome [12].

### Study participants

A convenience sample of patients of 65 years and older was selected. Patients were excluded when they were unable to speak or understand the Dutch language, had an acute life-threatening illness as judged by the attending physician or nurse, or had communication difficulties (e.g., severe hearing impairment).

### Translation

After permission of the original developer the English 4AT version 1.1 was translated into Dutch by use of guidelines for the process of cross-cultural adaption from Beaton et al. [13]. The original version was translated by two independent translators, an informed (MP) and uninformed translator (NS), to version T1 and T2. Any discrepancies were resolved and the two versions were synthesized in version T-12. A back translation was performed by a bilingual (first language) English speaker, version B1. Version B1 was sent to the original developer, with minor adjustments and approved for further use.

### Assessments

Each participant underwent two assessments; at first the index test 4AT, followed by the reference standard for delirium. A geriatric care professional; the team consists of geriatricians, internist-geriatricians, fellows internal medicine/geriatrics and nurse practitioners in geriatrics, performed the reference standard. The diagnosis of delirium was made with the Diagnostic and Statistical Manual of Mental Disorders (DSM-V) criteria [1]. The geriatric care professional performing the reference standard was blinded to results of the 4AT.

Demographic characteristics and comorbidity assessed by the Charlson Comorbidity Index (CCI) were registered [14, 15].

### Procedures

#### Emergency department

Patients were recruited during working hours. Research assistants (medical students, nurses) asked patients for participation. After verbal consent was obtained, the 4AT was administered. After completion of the 4AT, the research assistant contacted the attending geriatric expert and he/she assessed the patient in the ED. The time interval between both assessments was less than 4 h. The research assistant and geriatric expert were blinded to each other’s assessment. In a subgroup of the ED patients, the time to conduct the 4AT was measured. Afterwards, demographic characteristics were extracted from the Acutelines database.

#### Acute geriatric ward

Patients were recruited one day per week on the same day as the grand rounds took place. A research assistant (either a research nurse or physician) asked all eligible patients admitted to the ward for participation. After verbal consent was obtained, the 4AT was administered and demographic characteristics were collected by the research assistant. Afterwards, the attending physician of the ward, who was attending the grand rounds, was asked to register the presence or absence of delirium for every enrolled patient. The attending physician and research assistant were blinded to each other’s assessment. The time interval between the 4AT and the reference standard was less than 4 h.

### Statistical analysis

Demographic data were analyzed using descriptive statistics and are presented in means, standard deviations and percentages in case of a normal distribution and as medians and interquartile ranges (IQR) in case of a non-normal distribution. Diagnostic test accuracy was assessed by constructing a 2 × 2 table and sensitivity, specificity, positive predictive value (PPV), negative predictive value (NPV), and positive/negative likelihood ratios, along with 95% confidence intervals were calculated. Missing data regarding the index or reference test were excluded from the analysis. All analysis were carried out using SPSS 28 (IBM corp., Armonk, New York, USA).

## Results

In total, 71 patients in the acute geriatric ward were included and 49 patients were included in the ED. Table 1 shows the patient characteristics. The median (IQR) age of patients in the ED was 75 (72.5–79.5) years. The prevalence of delirium in the ED was 6.1%, while none of the ED patients had a known history of dementia. The median (IQR) age of patients in the acute geriatric ward was 83 (78–88) years. In the acute geriatric ward, the prevalence of delirium was 11.6%, while 16 (22.5%) patients had a known history of dementia.

**Table 1:** Patient characteristics

Characteristic	Emergency department *n* = 49	Acute geriatric ward *n* = 71
Age, median (IQR)	75 (72.5–79.5)	83 (78–88)
Female, *n* (%)	15 (30.6)	38 (53.5)
Charlson comorbidity index, median (IQR)	3 (1.5–6)	1 (1–2)
Known history of dementia, *n* (%)	0 (0)	16 (22.5)

*IQR* interquartile range

In the ED, 2 patients with a positive 4AT had also delirium according to the reference standard. For the acute geriatric ward, 7 patients with a positive test also had delirium according to the reference standard (Table 2). The sensitivity of the 4AT in the ED and acute geriatric ward was 0.67 (95% CI 0.09–0.99) and 0.88 (0.47–0.99) respectively (Table 3). The specificity was 0.83 (0.69–0.92) in the ED population and 0.69 (0.56–0.80) in the acute geriatric ward population. An additional subgroup analysis of patients with dementia was performed, but due to zero patients in the group with a negative 4AT and negative reference standard, results of this analysis did not contribute to assessment of the diagnostic test accuracy. Among the 16 patients with a known history of dementia in the  acute geriatric ward, 9 patients (56%) had a positive 4AT but not a delirium according to the DSM-criteria. These patients had median 7 (IQR 5–8) points on the 4AT, indicating possible delirium and/or cognitive impairment. In this small subgroup of 9 patients the AMT4, months backwards and acute change/fluctuation items contributed most often to 4AT score in patients with dementia but without delirium. Six patients with a known history of dementia had a negative 4AT and no delirium according to the DSM-criteria. One patient had a positive 4AT and had a delirium according to the DSM-criteria.

**Table 2:** 2 × 2 table

	Delirium present according to reference standard	Delirium absent according to reference standard
Emergency department		
4AT +	2	8
4AT −	1	38
Acute geriatric ward		
4AT +	7	19
4AT −	1	42

**Table 3:** Diagnostic test accuracy of the 4AT

	Sensitivity (95% CI)	Specificity (95% CI)	PPV	NPV	LR +	AUROC
4AT in ED	0.67 (0.09–0.99)	0.83 (0.69–0.92)	0.20	0.97	3.83	0.740
4AT in acute geriatric ward	0.88 (0.47–0.99)	0.69 (0.56–0.80)	0.27	0.98	2.81	0.799

*CI* confidence interval, *PPV* positive predictive value, *NPV* negative predictive value, *LR* + positive likelihood ratio, *AUROC* area under receiver operator curve, *ED* emergency department

In 23 patients in the ED the time needed to conduct the 4AT was measured. The median (IQR) time was 109 (80–147) seconds.

## Discussion

This study demonstrates the Dutch version of the 4AT is a reliable screening tool for delirium in both geriatric inpatients and older ED patients. Sensitivity for detecting delirium was higher in older patients admitted to the acute geriatric ward (88%), while specificity was higher in the ED setting (83%). The negative predictive value (NPV) of 0.97 in the ED setting and 0.98 in the acute geriatric ward setting means that respectively 97% and 98% of the patients with a negative 4AT did not have a delirium. This implies the Dutch version of the 4AT performs well to rule out the presence of delirium in the studied settings, similar to findings of the 4AT validation in other languages.[9, 16]. The lower positive predictive value (PPV) is as expected with a screening instrument, implying that a positive 4AT is not the same as the diagnosis of a delirium but should prompt the treating physician to further evaluate the presence of delirium [10].

The sensitivity of the 4AT in this study is similar to previous findings, however, the specificity is lower compared to validation studies of the 4AT in other languages, especially in the acute geriatric ward setting [17]. The finding of a lower specificity in the geriatric ward setting compared to the ED setting could be due to interference of other cognitive deficits (e.g., dementia) in the scoring of the 4AT and a higher prevalence of dementia in the acute geriatric ward setting than in ED setting. This is in line with previous studies showing dementia adversely affects the 4AT accuracy for delirium [7, 18, 19]. Furthermore, we made use of an unselected consecutive sample of ward patients, similar to Hendry et al. who reported a similar specificity of 70% [20]. Studies reporting a higher specificity often used more selected patient populations such as nursing homes and daily care centers in the Iranian version or the post-anesthesia care unit in the German version of the 4AT [21, 22]. Finally, as mentioned by Shenkin et al., the 4AT involves a degree of subjectivity. Alertness is scored in binary fashion; abnormal or normal in a period of minutes, while the reference standard assessment based on the DSM-V criteria is more detailed and relies on an observation of symptoms during 24 h This could also attribute to the high rate of false positives [8].

Our overall prevalence of delirium in the ED was lower than expected based on previous findings [2, 23, 24]. Possibly, there are multiple attributing factors to this lower than expected prevalence. In this study, the reference standard consisted of an assessment by a geriatric care professional and delirium was diagnosed according the Diagnostic and Statistical Manual of Mental Disorders (DSM-V) criteria, but in the ED information on the previous 24 h is sometimes lacking or incomplete. Other studies used different (additional) assessments such as checking a patient file, an additional psychiatric assessment or a screening instrument like the MMSE [18, 25, 26]. Another contributing factor could be a relatively young population of ED patients with median age of 75 years. Furthermore, there is a fluctuation in the course of delirium, with an observed difference between two assessments in the same patient because of change in mental state over time. As already stated by Han et al. a majority of ED patients with delirium will not have chief complaints of altered mental status and will be missed without actively looking for it [27]. We have tried to minimize the interference of time by setting an interval between 4AT and reference standard of maximal 4 h. The precise impact of the duration of the time interval between tests is not yet clear [10].

An acknowledged limitation of this study is the limited sample size and the fact a convenience sample was used. Occurrence of a selection bias, therefore, cannot be excluded which possibly correlates with the lower prevalence of delirium in comparison to other studies and might result in a lower generalizability. Nevertheless, we are confident the sample size is large enough to validate a translation of a test which has been extensively validated in different languages in the past decade [7, 10]. We consider the translation process as a strength of the study with the backtranslation being checked and approved by the original developer. The Dutch version of the 4AT corresponds well with the original English version. To assess any potential bias the QUADAS-2 criteria were used [28]. These criteria state estimates of test accuracy are based on the assumption that the reference standard is 100% sensitive and specific and any disagreements between the reference standard and index test are assumed to result from incorrect classification by the index test. Using the DSM-V criteria as the gold standard, which are based on subjective judgments of the assessor, a 100% sensitive and specific reference standard is not attainable and may influence the test results. Yet, the method used in this study is analogous to the used methods in previous studies on the diagnostic test accuracy of the 4AT, which makes it possible to compare study results.

The study of De (2016) showed nursing staff without dedicated training can successfully use the 4AT to screen for delirium [23]. The current study confirms this use and brevity of the Dutch version of the 4AT. This could be of use in making the emergency departments more senior-friendly. The regular ED staff could perform the screening and a health care professional with geriatric expertise can be involved in all patients with an abnormal score. The high sensitivity of the 4AT is useful, nobody will be missed. In a subgroup in this study, we found that conducting the 4AT by a non-trained assessor in the ED required less than 2 min. Therefore, this screening tool seems reliable and efficient even in a hectic and busy setting.

## Conclusion

In conclusion, this diagnostic accuracy study suggests the Dutch version of the 4AT is a valid screening tool for delirium detection in both acute geriatric wards and ED. Due to its brevity and practicality (i.e., no special training is required to administer the tool), it is useful in clinical practice.

## Data Availability

The data that support the findings of this study are not openly available but are available from the corresponding author upon request.
